# Phospho-Switch: Regulation of the Activity of SAM-Dependent Methyltransferases Using *H*-Phosphinic SAM Analogue

**DOI:** 10.3390/ijms26178590

**Published:** 2025-09-04

**Authors:** Vsevolod L. Filonov, Maxim A. Khomutov, Alexander Yu. Rudenko, Sofia S. Mariasina, Ratislav M. Ozhiganov, Alexander V. Sergeev, Sergei N. Kochetkov, Vladimir I. Polshakov, Elizaveta S. Gromova, Anastasia L. Khandazhinskaya, Alex R. Khomutov

**Affiliations:** 1 Engelhardt Institute of Molecular Biology, Russian Academy of Sciences, 119991 Moscow, Russia; filonov_vsevolod@mail.ru (V.L.F.); makhomutov@mail.ru (M.A.K.); snk1952@gmail.com (S.N.K.); khandazhinskaya@bk.ru (A.L.K.); 2 Belozersky Institute of Physico-Chemical Biology, Lomonosov Moscow State University, 119991 Moscow, Russiasofia.mariasina@yandex.ru (S.S.M.); lego-ratislav@yandex.ru (R.M.O.); 3 Faculty of Chemistry, Lomonosov Moscow State University, 119991 Moscow, Russia; avsergeev@belozersky.msu.ru (A.V.S.); vpolsha@mail.ru (V.I.P.); gromova@belozersky.msu.ru (E.S.G.); 4 Institute of Pharmacy and Biotechnology, RUDN University, 117198 Moscow, Russia; 5 Higher Chemical College RAS, Mendeleev University of Chemical Technology, 125047 Moscow, Russia

**Keywords:** S-adenosyl-L-methionine, H-phosphinic analogue of SAM, methyltransferases, COMT, DNA methylation, Dnmt1, Dnmt3a

## Abstract

S-Adenosyl-L-methionine (SAM) is a central cofactor in cellular methylation, donating methyl groups to a wide range of biological substrates. SAM analogues are promising tools for selective modulation of methyltransferase activity. Here, we investigated phosphorus-containing analogues of SAM and S-adenosyl-L-homocysteine (SAH), focusing on the *H*-phosphinic SAM analogue ((R,S)-SAM-P_H_) with the HO(H)(O)P group replacing the carboxyl group of SAM. We examined the interaction of (R,S)-SAM-P_H_ with three representative methyltransferases: Dnmt1, responsible for maintenance of DNA methylation; Dnmt3a, which establishes de novo DNA methylation; and catechol-*O*-methyltransferase (COMT), which methylates protocatechuic aldehyde to yield vanillin and isovanillin. (R,S)-SAM-P_H_ is a methyl group donor for Dnmt3a and COMT, but not for Dnmt1, despite the high structural similarity of the Dnmt1 and Dnmt3a catalytic domains. These results demonstrate that targeted modification of the carboxyl group of SAM can yield analogues with specific activity towards various methyltransferases. The different recognition of (R,S)-SAM-P_H_ by Dnmt3a and Dnmt1 highlights its potential as a molecular probe for distinguishing de novo from maintenance DNA methylation. This work enriches our understanding of methyltransferase substrate specificity and provides a new tool for selective modulation of epigenetic processes.

## 1. Introduction

S-Adenosyl-L-methionine (SAM) ranks second only to ATP in terms of the diversity of biochemical transformations it participates in. SAM functions as a donor of methyl, aminocarboxypropyl, and adenosyl groups, which are transferred to nucleic acids, proteins, and various low-molecular-weight compounds [1,2,3,4]. In its decarboxylated form, SAM also serves as a donor of the aminopropyl group in the biosynthesis of the biogenic polyamines spermine and spermidine [5]. SAM-dependent radical reactions are widespread, including the methylation of inert substrates, particularly at carbon or phosphorus atoms [6].

In addition to its role as a donor molecule, SAM also acts as an allosteric regulator of enzymes, such as cystathionine β-synthase [7] and methylenetetrahydrofolate reductase [8]. In plants, the growth hormone ethylene is synthesized from SAM via the intermediate 1-aminocyclopropane-1-carboxylic acid [9]. However, the majority of SAM synthesized in cells is consumed in enzymatic methylation reactions, which are crucial for the regulation of the activity and functions of DNA, RNA, proteins, and small molecules [2].

Chemical regulation of SAM-dependent reactions represents a valuable strategy for the design of biologically active compounds [10]. Given the diversity of methyltransferase reactions, the existence of a universal inhibitor is unlikely. Nonetheless, sinefungin (5′-adenosyl-L-ornithine), an antibiotic, is one of the most broadly acting inhibitors. It competes with SAM for reversible binding to the active sites of methyltransferases [2,11].

Inhibitors of DNA methyltransferases have antitumor activity, partly due to their ability to reactivate silenced tumour suppressor genes [12]. Among these, azacitidine and decitabine are the most well-known and widely used in oncology. These nucleoside analogues incorporate into DNA, replacing cytosine with 5-azacytosine. This leads to the formation of a covalent bond between the carbon-6 atom of the modified cytosine and DNA methyltransferases, resulting in a stable enzyme-DNA complex and inhibition of methyltransferase activity [13]. Notably, azacitidine also functions as a dual-target inhibitor by activating interferon-dependent signalling pathways in ovarian cancer cells [14]. The combined use of azacitidine and α-difluoromethylornithine, an irreversible inhibitor of ornithine decarboxylase, the rate-limiting enzyme in polyamine biosynthesis, demonstrated superior efficacy in vitro and in vivo in an ovarian cancer mouse model compared to either agent alone. This underscores the potential of multi-target therapies involving epigenetic regulators [15].

During methyltransferase-catalyzed reactions, SAM is converted into S-adenosyl-L-homocysteine (SAH), a potent inhibitor of most methyltransferases. Thus, the SAM/SAH ratio serves as a key indicator of cellular methylation efficiency [16]. Inhibitors of SAH hydrolase lead to intracellular SAH accumulation, altering this ratio and exerting strong antiviral effects, primarily by inhibiting the maturation of viral mRNA via interference with 5′-cap formation [17]. Amplification of the SAH hydrolase gene has been observed in several malignancies, suggesting it as a potential therapeutic target [18]. Indeed, SAH hydrolase inhibitors such as neplanocin A, 3-deazaneplanocin A, and aristeromycin exhibit antitumor activity both in vitro and in vivo [19,20,21].

An alternative approach to modulating SAM-dependent processes is the use of SAM analogues that either function as less effective substrates for methyltransferases or selectively target specific enzymes.

**Figure 1:**
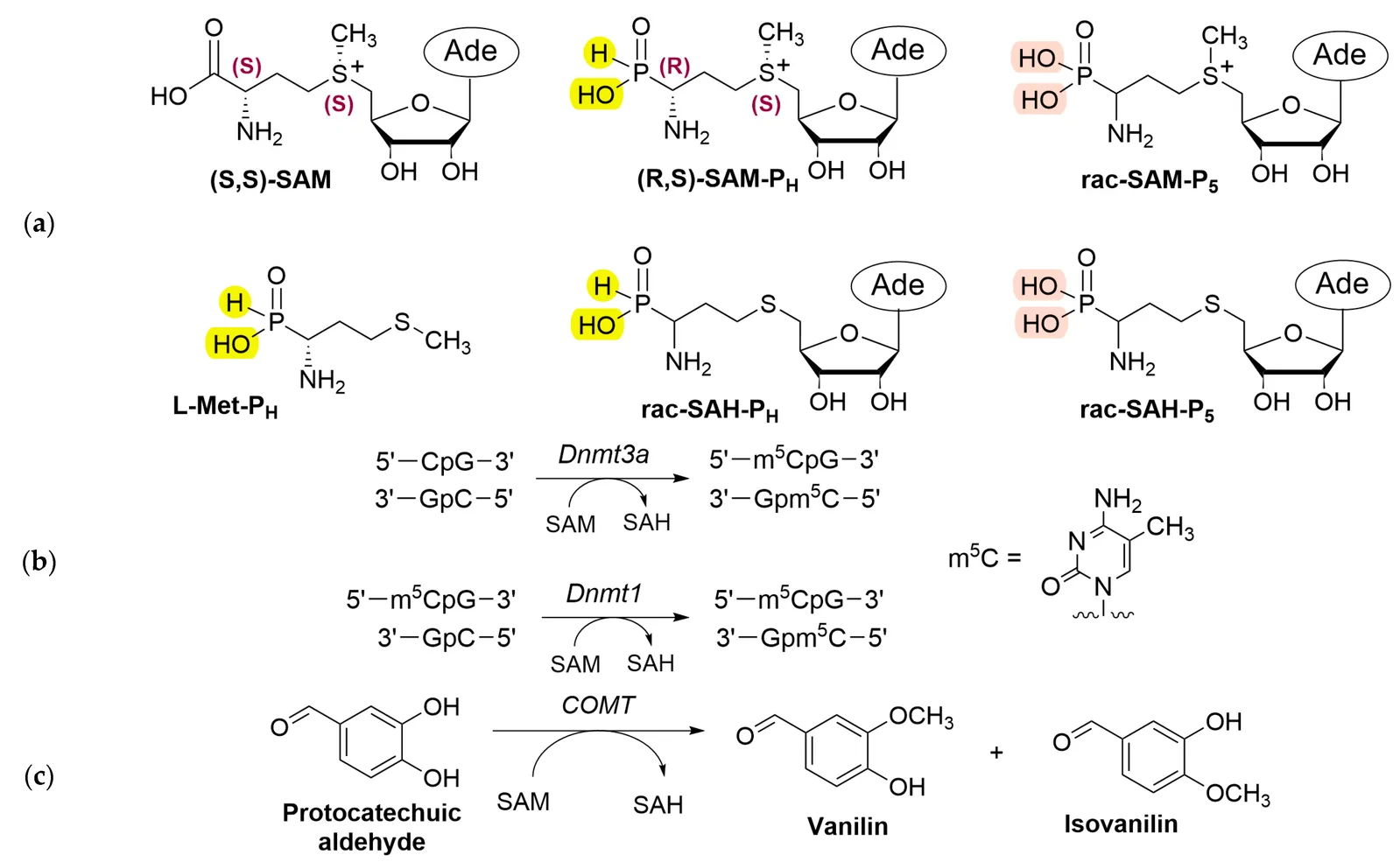
Phosphorus-containing analogues of methyltransferase substrates/inhibitors and selected reactions catalyzed by these enzymes. (**a**) Structures of the H-phosphinic analogue of methionine (L-Met-P_H_), (S,S)-SAM, *H*-phosphinic analogues of SAM and SAH ((R,S)-SAM-P_H_ and rac-SAH-P_H_), and phosphonic analogues of SAM and SAH (rac-SAM-P_5_ and rac-SAH-P_5_). H-Phosphinic and phosphonic groups are marked in yellow and rose, respectively. Individual diastereomers have hashed wedge bonds at the chiral centres, while the corresponding racemates are depicted using solid bonds. (**b**) DNA-(cytosine C5) methylation at CpG sites catalyzed by Dnmt3a, which establishes de novo DNA methylation patterns [24], and methylation at hemimethylated CpG sites catalyzed by Dnmt1, which maintains DNA methylation patterns during replication in mammals [25]. (**c**) Methylation of protocatechuic aldehyde catalyzed by catechol-O-methyltransferase (COMT).

Recently we chemically synthesized racemic phosphonic and H-phosphinic analogues of SAM—rac-SAM-P_5_ and rac-SAM-P_H_, respectively (Figure 1a)—as well as the corresponding phosphorus-containing analogues of SAH—rac-SAH-P_5_ and rac-SAH-P_H_ [22]. These SAM analogues demonstrated significantly greater stability than natural SAM, making them valuable tools for investigating methyltransferase activity. For instance, at pH 8.0, the half-life of rac-SAM-P_H_ is 55 h, compared to just 11 h for SAM [22].

Importantly, (R,S)-SAM-P_H_—with stereochemistry identical to natural SAM—was successfully synthesized enzymatically from the *H*-phosphinic analogue of methionine (L-Met-P_H_, Figure 1a) using SAM synthetase (MAT2A) or from SAH-P_H_ via halomethyltransferase [22]. We recently demonstrated that in the de novo DNA methylation reaction catalyzed by Dnmt3a, (R,S)-SAM-P_H_ was only two-fold less efficient as a methyl donor than natural SAM [23].

In the present study, we performed a comparative analysis of the interaction of SAM and SAH phosphorus-containing analogues with DNA methyltransferases Dnmt1 and Dnmt3a (Figure 1b), as well as with catechol-O-methyltransferase (COMT), using the methylation of protocatechuic aldehyde to form vanillin and isovanillin as a model reaction (Figure 1c). Our findings show that (R,S)-SAM-P_H_ is a good substrate for Dnmt3a, is approximately five times less efficient than SAM as a COMT substrate, and is not a substrate for Dnmt1. These results reveal promising and selective opportunities for chemical regulation of biomethylation processes.

## 2. Results

### 2.1. Phosphorus-Containing Analogues of SAM: Substrates for Dnmt3a and COMT, but Not Dnmt1

Among the established Dnmt1 activity assay protocols, the D1 DNA hairpin assay is particularly attractive, as it eliminates the need for radioactively labelled SAM [26]. In the original protocol, methylation and cleavage were performed in one pot using a reaction mixture containing model hemimethylated DNA hairpin D1 (30 nM), SAM (1 mM), Dnmt1 (2.8 nM), methyl-dependent endonuclease GlaI (0.05 U per 1.0 µL of reaction mixture), and MgCl_2_ (5 mM). Under these conditions, DNA methylation reached only 40% after 8 h [26].

**Figure 2:**
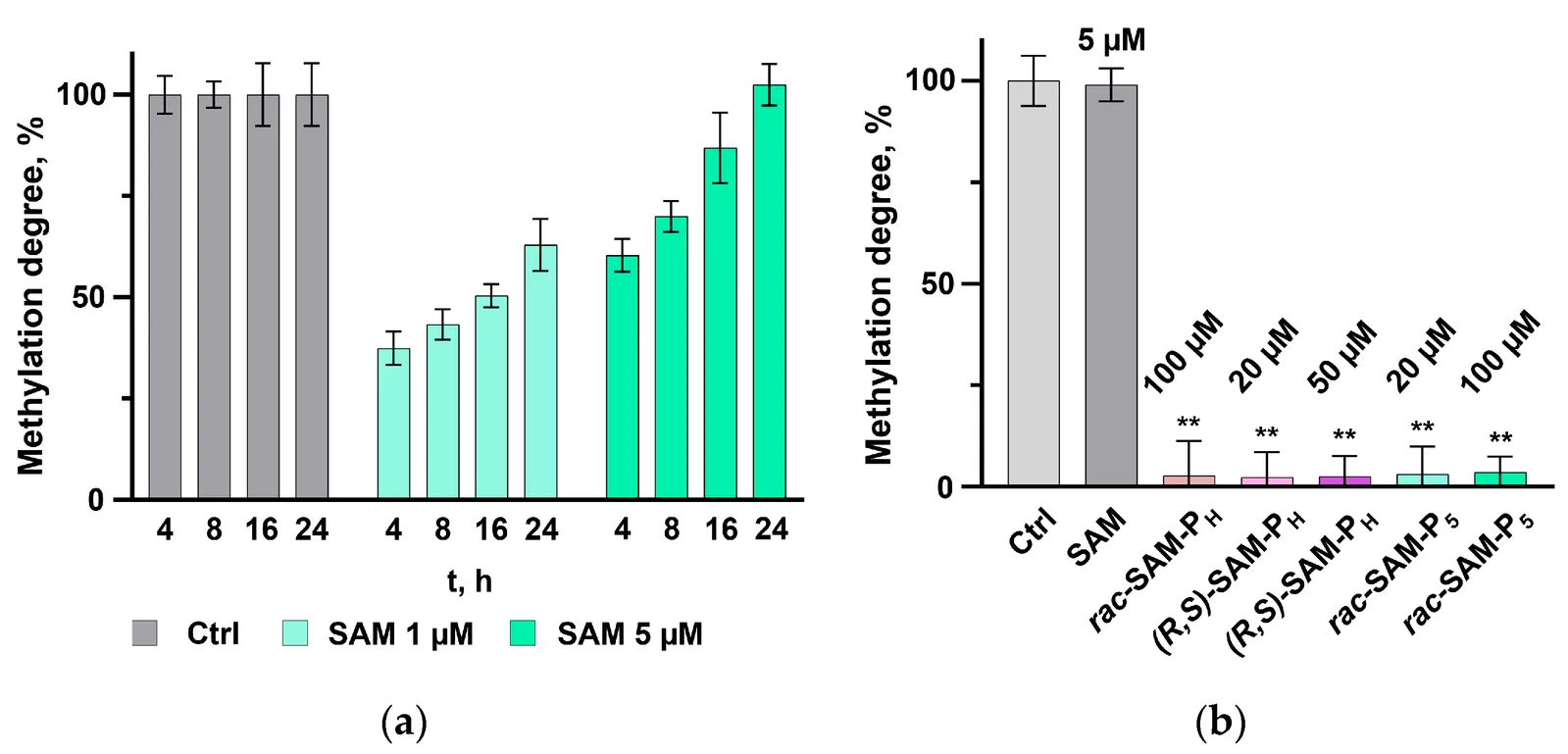
Effect of SAM phosphorus-containing analogues on Dnmt1 activity. (**a**) Methylation of D1 hairpin DNA fragment by Dnmt1 in a time-dependent and SAM concentration-dependent manner at 37 °C for 4–24 h using 1.0 µM and 5.0 µM SAM. (**b**) Dnmt1 methylation activity (24 h) in the presence of SAM phosphorus-containing analogues: rac-SAM-P_H_ (100 µM), (R,S)-SAM-P_H_ (20 µM and 50 µM), or rac-SAM-P_5_ (20 µM and 100 µM), compared with SAM (5 µM). In both panels, the control was D2 hairpin DNA (a product of methylation) digested with GlaI. In both panels the results indicate means from five independent experiments. Error bars represent standard deviations. ** *p* ≤ 0.01 according to one-way ANOVA with Dunnett’s post hoc test.

We improved this method by separating the methylation and cleavage steps. During the methylation stage, the Dnmt1 concentration was increased to 10 nM, MgCl_2_ was omitted, and the SAM concentration was reduced to 5 µM. For the subsequent cleavage step, MgCl_2_ (5 mM) and GlaI (0.05 U per 1.0 µL of reaction mixture) were added. Under these optimized conditions, DNA methylation developed in time and reached 100% after 24 h (Figure 2a). In contrast, when the SAM concentration was reduced to 1 µM, only 60% methylation was observed after 24 h (Figure 2a). SAM was not replenished in these reactions, and incomplete methylation when using 1 µM SAM may be due to its instability at neutral and slightly alkaline pH [27]. When using 5 µM SAM, this decomposition is not so essential. It should be noted that SAM-P_H_ is more stable at neutral pH (τ ½ is about 55 h at pH 8.0 and 37 °C [22]) and no replenishment is needed.

Notably, when 100 µM of rac-SAM-P_H_ or rac-SAM-P_5_ were used as methyl group donors, only little, if any, DNA methylation occurred after 24 h (Figure 2b). The lack of the activity of rac-SAM-P_H_ and rac-SAM-P_5_ is unlikely to be explained by the presence of inhibitory diastereomers in the racemic mixtures. (R,S)-SAM-P_H_ (ee approximately 97–98%, Figure S1) has the same stereoconfiguration as natural SAM (Figure 1a) but was not a substrate of Dnmt1 (Figure 2b). The incorporation of the methyl group into the hemimethylated D1 DNA hairpin was approximately at the same 2–4% using either rac-SAM-P_H_ (100 μM) or (R,S)-SAM P_H_ (50 μM) as cofactors (Figure 2b). Taking into consideration the activity assay method, these 2–4% of the activity are within the experimental error. Thus, neither (R,S) SAM P_H_ nor rac-SAM-P_5_ are the substrates of Dnmt1 in contrast with Dnmt3a.

In contrast, analysis of the interaction between SAM phosphorus-containing analogues and Dnmt3a revealed that both (R,S)-SAM-P_H_ (5 µM) and rac-SAM-P_5_ (20 µM) function as methyl group donors, exhibiting approximately half the potency of natural SAM at the same concentration (5 µM) (Table 1). Notably, (R,S)-SAM-P_H_ also proved to be a substrate for catechol-*O*-methyltransferase (COMT): methylation of protocatechuic aldehyde reached 22% after 2 h, whereas complete methylation was observed with natural SAM under the same conditions. In contrast, rac-SAM-P_5_ did not exhibit substrate activity toward COMT (Table 1).

**Table 1:** Phosphorus-containing analogues of SAM ((R,S)-SAM-P_H_ and rac-SAM-P_5_) as donors of methyl groups in the reactions catalyzed by Dnmt1, Dnmt3a, and COMT. The activity of Dnmt1 was determined as described above, and the activities of Dnmt3a and COMT were determined according to those described in [22,23], respectively. Methylation degrees are given relative to SAM. The murine catalytic domain of Dnmt3a (Dnmt3a-CD) was used [23].

Cofactor	Methylation Activity, %		
	Dnmt1	Dnmt3a	COMT
SAM	100	100	100
(R,S)-SAM-P_H_	Not a donor of methyl group	59 [23]	22 *
rac-SAM-P_5_	Not a donor of methyl group	51 [23]	Not a donor of methyl group

* methylation degree after 2 h.

These findings highlight (R,S)-SAM-P_H_ as a novel chemical probe allowing discrimination of de novo and maintenance DNA methylation. Differences in (R,S)-SAM-P_H_ substrate properties to Dnmt3a and Dnmt1 offer an alternative to the strategies using selective enzyme inhibitors. For example, the bisubstrate quinazoline–quinoline inhibitor exhibits approximately 100-fold selectivity for Dnmt3a over Dnmt1 [28], while certain *N*-alkyl tryptamine derivatives preferentially inhibit Dnmt1 with up to 20-fold selectivity over Dnmt3a [29].

### 2.2. Inhibition of Dnmt1 by SAM and SAH Phosphorus-Containing Analogues

**Figure 3:**
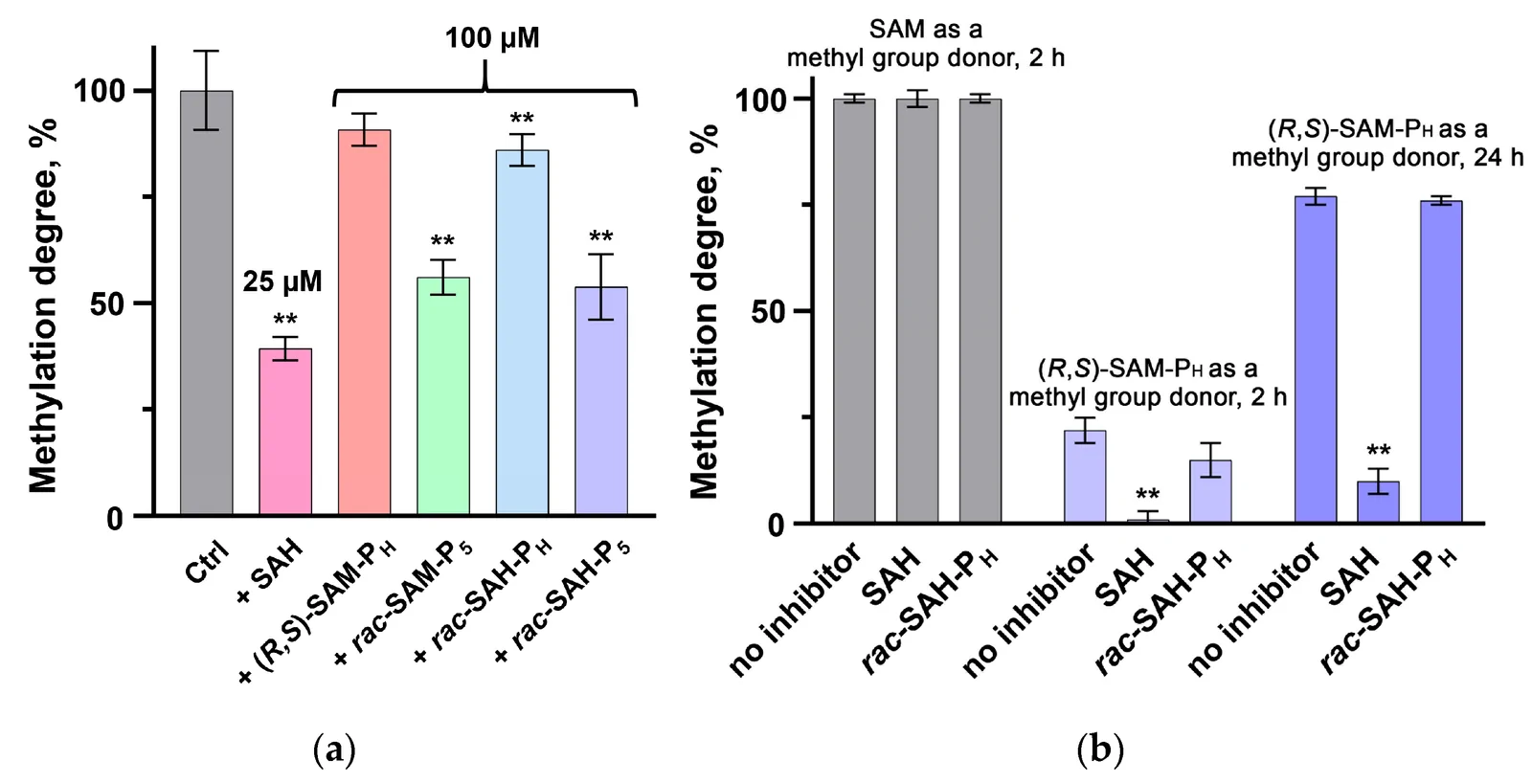
Interaction of SAM and SAH phosphorus-containing analogues with Dnmt1 and COMT. (**a**) Inhibition of Dnmt1 methylation activity. Dnmt1 reaction mixtures contained the following: SAM (5 µM), rac-SAH-P_5_ (100 µM), rac-SAM-P_5_ (100 µM), rac-SAH-P_H_ (100 µM), or (R,S)-SAM-P_H_ (100 µM). SAH (25 µM) was used as a reference inhibitor. The D2 DNA hairpin, the product of the Dnmt1 reaction cleaved with GlaI, was used as the control. (**b**) Inhibition of COMT methylation activity. Reaction mixtures contained either SAM or (R,S)-SAM-P_H_ (each 6 mM) as methyl group donors and SAH (3 mM) or rac-SAH-P_H_ (3 mM) as inhibitors. The duration of the reactions was 2 h and 24 h. In both panels the results indicate means from five independent experiments. Error bars represent standard deviations. ** *p* ≤ 0.01 according to one-way ANOVA with Dunnett’s post hoc test (vs. “no inhibitor”).

Since (R,S)-SAM-P_H_ and *rac*-SAM-P_5_ were not substrates of Dnmt1 (Figure 2b), it remained unclear whether these analogues could compete with SAM for binding to the enzyme. To investigate this, inhibition assays were performed by adding 100 µM of each SAM phosphorus-containing analogue to reaction mixtures containing 5 µM SAM. Methylation activity was measured after 24 h. Under these conditions, rac-SAM-P_5_ inhibited Dnmt1 for 30–40%, whereas (R,S)-SAM-P_H_ showed no significant inhibitory activity (Figure 3a).

Given that SAH is a natural inhibitor of most methyltransferases, including Dnmt1, the potential inhibitory effects of rac-SAH-P_H_ and rac-SAH-P_5_ were also evaluated using the same experimental setup: 100 µM SAH phosphorus-containing analogues were added to reactions containing 5 µM SAM. It was found that rac-SAH-P_5_ inhibited Dnmt1 by approximately 40%, while rac-SAH-P_H_ was even less effective and inhibited the enzyme for only 10–15% (Figure 3a). For comparison, SAH at 25 µM inhibited Dnmt1 by 60% under the same conditions (Figure 3a). Based on these results, neither rac-SAM-P_5_ nor rac-SAH-P_5_ exhibited sufficient potency, and their inhibitory activity was not investigated further.

Dnmt3a, unlike Dnmt1, accepted (R,S)-SAM-P_H_ and rac-SAM-P_5_ as substrates (Table 1), while both rac-SAH-P_H_ and rac-SAH-P_5_, taken at 5 µM, inhibited Dnmt3a for 70% and 50%, respectively, in reactions containing 1 µM SAM [23]. In comparison, natural SAH (5 µM) completely inhibited Dnmt3a under the same conditions [23].

The COMT-catalyzed reaction was monitored by ^1^H NMR spectroscopy (Figure S3), which required the use of high concentrations of the enzyme, SAM, and (R,S)-SAM-P_H_. When natural SAM (6 mM) was the methyl group donor (2 h), neither SAH (3 mM) nor rac-SAH-P_H_ (3 mM) inhibited the COMT reaction (Figure 3b). When (R,S)-SAM-P_H_ (6 mM) was the methyl group donor (2 h), natural SAH (3 mM) completely inhibited the reaction (2 h), but rac-SAH-P_H_ (3 mM) was inactive (Figure 3b). SAH (3 mM) inhibits the reaction for about 80% when (R,S)-SAM-P_H_ (6 mM) was the methyl group donor, while rac-SAH-P_H_ (3 mM) did not inhibit the COMT reaction (24 h). These findings are consistent with the lower affinity of (R,S)-SAM-P_H_ for COMT compared to natural SAM.

Phosphorus-containing analogues of SAM and SAH exhibit distinct patterns of interaction with Dnmt1, Dnmt3a, and COMT: (i) (R,S)-SAM-P_H_ functions as a methyl group donor in the Dnmt3a-mediated DNA methylation and in the COMT-mediated methylation of protocatechuic aldehyde, but is not a substrate for Dnmt1; (ii) rac-SAM-P_5_, like (R,S)-SAM-P_H_, serves as a substrate for Dnmt3a. However, it does not display substrate or inhibitory activity in the COMT reaction and, unlike (R,S)-SAM-P_H_, acts as a weak competitive inhibitor of Dnmt1; (iii) rac-SAH-P_H_ and rac-SAH-P_5_ both inhibit Dnmt3a to a similar extent, though less effectively than natural SAH. In the case of Dnmt1, only rac-SAH-P_5_ exhibits weak inhibitory activity, whereas for COMT, inhibition is observed only with SAH-P_H_, which shows modest activity.

Thus, among the phosphorus-containing analogues of SAM and SAH (Figure 1a), (R,S)-SAM-P_H_ had the most interesting set of properties, which makes it a promising new research tool that allows discrimination between different methyltransferase reactions.

## 3. Discussion

**Figure 4:**
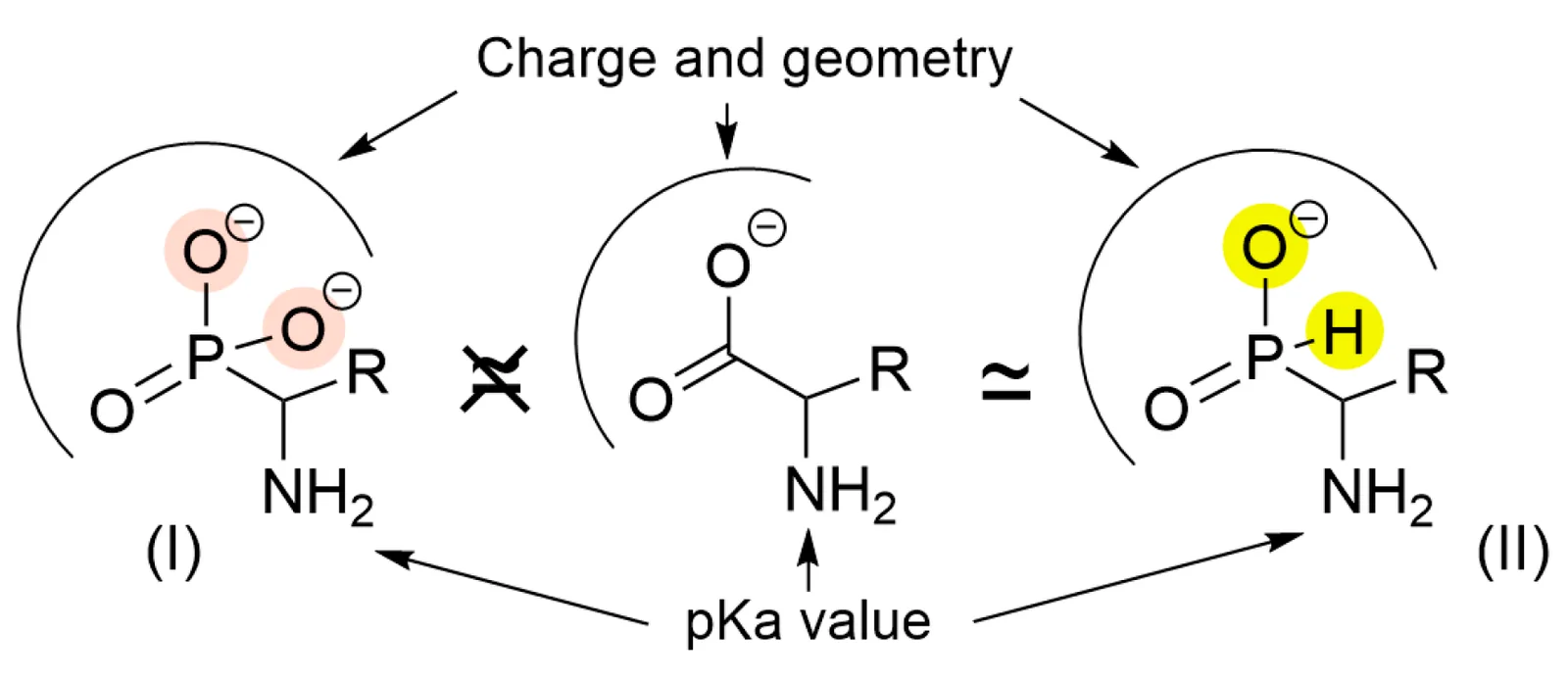
Structures of α-aminocarboxylic acids, α-aminophosphonic acids (I), and α-amino-*H*-phosphinic acids (II). Key difference between α-aminophosphonic acids (I), and α-amino-*H*-phosphinic acids (II) are highlighted. *H*-Phosphinic and phosphonic groups are marked in yellow and rose, respectively.

Substitution of the carboxyl group in amino acids with an acidic phosphorus-containing moiety yields several classes of analogues, notably α-aminophosphonic (I) and α-amino-*H*-phosphinic (II) acids (Figure 4).

α-Aminophosphonic acids (I, Figure 4) typically are weak competitive inhibitors of enzymes involved in amino acid metabolism [30]. This limited efficacy arises from the phosphonic group’s bulky, double-charged tetrahedral structure, which poorly mimics the planar geometry of the single-charged carboxyl group. However, there are some exceptions: for instance, the phosphonic analogue of alanine (R = CH_3_) irreversibly inhibits alanine racemase [31], and the SAH phosphonic analogue (SAH-P_5_; Figure 1a) inhibits SAH hydrolase irreversibly [32].

The *H*-phosphinic group (II, Figure 4) is a single-charged, flattened tetrahedron, which more closely resembles the planar geometry of the single-charged carboxyl group and is its bioisostere [33]. The pKa of the amino group in α-amino-*H*-phosphinic acids (II, Figure 4) is approximately one unit lower if compared with natural amino acids [34], a property important for productive substrate binding. Taken together, these features and properties allow α-amino-*H*-phosphinic acids to participate in enzymatic reactions as substrate mimics, yielding novel compounds with carbon–phosphorus–hydrogen (*C–P–H*) bonds.

For example, pyridoxal 5’-phosphate (PLP)-dependent alanine and aspartate transaminases convert α-amino-*H*-phosphinic analogues of alanine (R = CH_3_), aspartate (R = CH_2_COOH), and glutamate (R = CH_2_CH_2_COOH) into the corresponding α-keto-*H*-phosphinic acids [35,36]. Similarly, PLP-dependent enzymes such as tyrosine phenol-lyase and methionine γ-lyase cleave *H*-phosphinic analogues of tyrosine (R = CH_2_C_6_H_4_OH) and methionine (R = CH_2_CH_2_SCH_3_) to produce *H*-phosphinic analogues of pyruvate [37] and α-ketobutyrate [38], respectively.

In this study, we examined the interaction of SAM and SAH *H*-phosphinic analogues ((R,S)-SAM-P_H_ and rac-SAH-P_H_, respectively; Figure 1a) with selected methyltransferases: Dnmt1 which maintains DNA methylation during replication; Dnmt3a, which establishes methylation patterns in mammals (Figure 1b); and catechol-O-methyltransferase (COMT), which methylates mammalian catechol-based neurotransmitters (Figure 1c).

Recently, using the distal *H*-phosphinic analogue of glutamate (Glu-γ-P_H_), we demonstrated that the *H*-phosphinic group at the γ-position to the reaction centre does not hinder enzymatic transformation at the amino acid part of the molecule. For example, *L*-Glu-γ-P_H_ is converted by PLP-dependent glutamate decarboxylase into the *H*-phosphinic analogue of γ-aminobutyric acid (GABA-P_H_), which is subsequently transaminated to the *H*-phosphinic analogue of succinic semialdehyde. This compound is then oxidized by NAD^+^-dependent succinic semialdehyde dehydrogenase to yield the *H*-phosphinic analogue of succinate [33]. Similarly, *L*-Glu-γ-P_H_ undergoes oxidative deamination by NAD^+^-dependent glutamate dehydrogenase—this is another example of the transformation taking place at the γ-position to the *H*-phosphinic group [39].

Despite the centrality of SAM in methyltransferase reactions, the impact of the carboxyl group modifications on the substrate properties of the corresponding derivatives is poorly studied, especially for eukaryotic enzymes. For example, methyl and ethyl esters of SAM have been shown to act as methyl donors for the CpG-specific prokaryotic cytosine-C5 DNA methyltransferase M.SssI only marginally less effective than natural SAM [40]. These esters are also accepted by COMT [40]. However, to the best of our knowledge, the interaction of SAM analogues with modified carboxyl groups with eukaryotic DNA methyltransferases has never been studied yet.

Our data reveal that methyltransferases exhibit distinct interactions with (R,S)-SAM-P_H_ (Figure 1). This analogue serves as a competent methyl donor in the Dnmt3a-CD-catalyzed DNA methylation reaction [23] but is a worse substrate in the COMT-mediated methylation of protocatechuic aldehyde (Table 1). Unexpectedly, Dnmt1 neither recognizes (R,S)-SAM-P_H_ as a substrate (Figure 2b) nor as an inhibitor (Figure 3a).

Dnmt1 and Dnmt3a each contain C-terminal catalytic and N-terminal regulatory domains [41,42]. Their C-terminal domains share high sequence similarity to well-studied prokaryotic C5 DNA methyltransferases, such as M.HhaI, includes ten conserved motifs responsible for SAM binding (motifs I and X) and catalysis (motifs IV, VI, and VIII), suggesting a conserved mechanism of action [43]. Only the target recognition domain (TRD) inside the catalytic domain of Dnmt1 is exceptionally long compared to those in other DNA methyltransferases [44].

Catalytic activity of Dnmt1 and Dnmt3a is under allosteric control of N-terminal domains with autoinhibitory function, the RFT and CXXC domains in Dnmt1, and the ADD domain in Dnmt3a [45]. The C-Terminal domain of full-length Dnmt1 is catalytically inactive and does not act as an independent methyltransferase [46]. The structural studies demonstrated that the proper folding of the catalytic domain likely requires its N-terminal domains [46]. So, the interaction of the N-terminal part with the C-terminal domain is necessary to activate the enzyme for methylation of hemimethylated substrates [47].

In contrast, the catalytic domain of Dnmt3a, i.e., Dnmt3a-CD, is active in an isolated form and has been used as a model system to study the catalytic mechanism and specificity of the enzyme [46,47]. However, these differences between Dnmt1 and Dnmt3a may hardly explain the differences in the interaction of these enzymes with the phosphorus-containing analogues of SAM and SAH.

In sum, the full-length Dnmt1 and Dnmt3a-CD in our assays provide functionally competent forms in vitro and can be used for studying the properties and activities of novel substrate-like (R,S)-SAM-P_H_.

Dnmt3a does not exhibit a strong preference for unmethylated vs. hemimethylated CpG sites. In vivo and in vitro, Dnmt3a forms an active heterotetramer (Dnmt3L–Dnmt3a–Dnmt3a–Dnmt3L), with enzymatically inactive regulatory factor Dnmt3L [47]. Dnmt3a can form an active homotetramer in the absence of Dnmt3L.

Dnmt1 ensures the copying of the methylation pattern onto the growing chain and is associated with the replication fork. The substrate of the enzyme is hemimethylated CpG sites of DNA. The specificity of Dnmt1 towards hemimethylated DNA arises from autoinhibition triggered by binding to unmethylated DNA, which prevents de novo methylation [44]. The necessity of contacts between different domains of the N-terminal part of Dnmt1 and its C-terminal catalytic domain explains why the isolated terminal domain of Dnmt1 does not have catalytic activity [41]. These differences are in line with the observed differences in the interaction of phosphorus-containing analogues SAM with full-length Dnmt1 and the catalytic domain of Dnmt3a.

It is known that the catalytic domains of Dnmt1 and Dnmt3a have a high degree of homology [42]. The key amino acid residues of Dnmt1 and Dnmt3a responsible for the cofactor binding are conserved; sequence alignment of the C-terminal domains of these mammalian enzymes shows that there are some variable residues, which may generate different interactions with the cofactor [48,49]. Accordingly, structural alignment obtained by molecular simulations indicates that there are subtle differences beyond the canonical motifs that could affect recognition of SAM and its analogues. The cofactor-binding pocket of the human Dnmt1 contains, among others, the variable Asn1578 and Val1580, which in Dnmt3a are changed to charged Arg891 and bulkier Trp893, respectively [49]. These Arg and Trp residues bind the HOOC group of SAH in the complex with Dnmt3a [48], and it may be assumed that the same residues are involved in binding the *H*-phosphinic group of (R,S)-SAM-P_H_. In the case of Dnmt1, the HOOC group of SAH is surrounded by Gly1149, Gly1150, Leu1151, and Val1580 [48]. Most likely, these amino acid residues cannot properly anchor the bulky *H*-phosphinic group, which has a flattened tetrahedral conformation, in contrast to the planar HOOC group. These differences in the structural organization of the cofactor-binding pockets of Dnmt3a and Dnmt1 might explain why (R,S)-SAM-P_H_ is the substrate of the former but not the latter DNA methyltransferase.

Moreover, murine and human Dnmt1 bind SAM differently in the absence of DNA [50]. Under these conditions, the catalytically important cysteine residue of murine Dnmt1 (in the PCQ loop of motif IV) is oriented similarly to that in the complex with DNA, whereas in human Dnmt1 in the absence of DNA, the corresponding cysteine residue is only partially aligned with the active site [50]. Thus, even two orthologous methyltransferases recognize natural SAM differently.

Unlike most enzymes of amino acid metabolism, Dnmt3a exhibits a highly atypical interaction pattern with phosphorus-containing substrate analogues. Both rac-SAM-P_H_ and rac-SAH-P_5_ were only about twofold less effective substrates of Dnmt3a than natural SAM [23]. In contrast, COMT conforms to the conventional behaviour observed for the enzymes of amino acid metabolism: (R,S)-SAM-P_H_ exhibited roughly fivefold lower activity than SAM, while rac-SAH-P_5_ was neither a substrate nor an inhibitor of the enzyme [22].

Our data confirm that (R,S)-SAM-P_H_ interacts differentially with Dnmt1, Dnmt3a, and COMT (Table 1). This offers a novel strategy for the selective chemical modulation of methyltransferase activity. Rather than inhibition, selectivity is achieved through differential substrate recognition of the SAM analogue.

## 4. Materials and Methods

The amino acid parts of SAM, as well as SAM-P_H_, contain two chiral centres. Therefore, rac-SAM-P_H_, chemically synthesized according to [22], is a mixture of (R,R)-, (S,S)-, (S,R)-, and (R,S)-diastereomers. The signals of the methyl group of the sulfonium centre having either (R)- or (S)-configurations are clearly visible in the ^1^H-NMR spectrum (see Figure S1). Characteristic signals of all four diastereomers are separated in the ^13^C-NMR spectrum (see Figure S2). In contrast, (R,S)-SAM-P_H_, enzymatically synthesized according to [22], has a stereoconfiguration the same as natural SAM and is enantiomerically pure (according to NMR ee > 97–98%); see Figures S1 and S2. The rest of phosphorus-containing analogues of SAM and SAH: rac-SAM-P_H_, (R,S)-SAM-P_H_, rac-SAM-P_5_, rac-SAH-P_H_ and *rac*-SAH-P_5_ (Figure 1a) were synthesized according to the protocol published in [22]. SAM and SAH were purchased from Sigma (CШA); all of the other reagents were of the highest purity available.

Methyl-dependent endonuclease GlaI (recognition site: Gm^5^CGm^5^C/Gm^5^CGm^5^C) was purchased from SibEnzyme (Novosibirsk, Russia). Human recombinant DNA methyltransferase Dnmt1 was purchased from ThermoFisher (Waltham, MA, USA). Human recombinant catechol *O*-methyltransferase (COMT) cysteine-free mutant C33S, C69V, C95S, C157S, C173S, C188R, and C191A were prepared according to the protocol described in [22].

Trimethylated DNA hairpin D1 and tetramethylated DNA hairpin D2, modified with fluorophore Cyanine 3 (Cy3) and fluorescence quencher Black Hole Quencher 2 (BHQ2), were purchased from «DNA-synthesis» (Russia).

D1: 5′-Cy3-CCTATGCGm^5^CATCAGTTTTCTGATGm^5^CGm^5^CATAGG-BHQ2-3′

D2: 5′-Cy3-CCTATGm^5^CGm^5^CATCAGTTTTCTGATGm^5^CGm^5^CATAGG-BHQ2-3′

Fluorescence measurement (assay of Dnmt1 activity) was performed in 96-well microplates Greiner 96 Flat Black, Cat. No. 655,090 (Boree Creek, Austria) using microplate reader Spark 10M (Vienna, Austria): λ_ex_ 543 nm, λ_em_ 563 nm, gain 240, Z-position 19,000 µm, integration time 40 µs.

Buffer A contained 10 mM Tris-HCl (pH 7.5), 5% glycerol, 100 mM NaCl, 0.1 mg/mL BSA, and 1 mM DTT.

### 4.1. Dnmt1 Activity Assay, Substrate Properties of Phosphorus-Containing Analogues of SAM and Inhibitory Activity of Phosphorus-Containing Analogues of SAH

**Figure 5:**
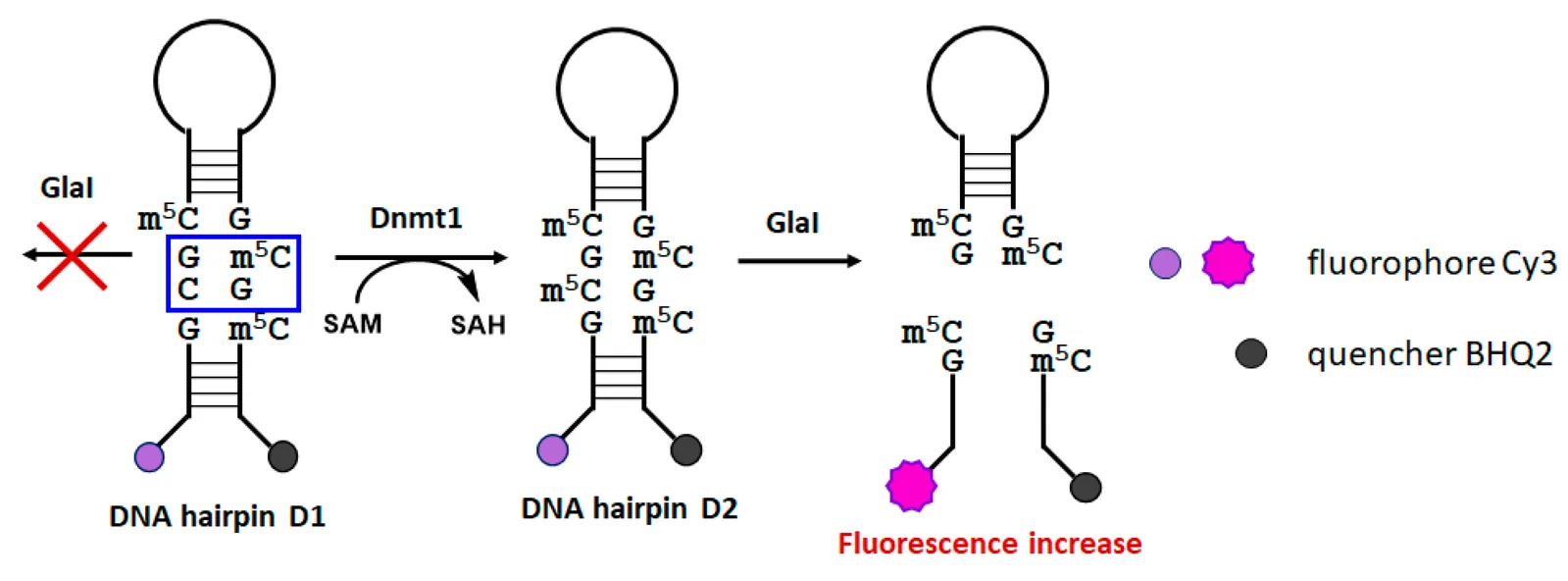
Dnmt1 activity assay using a hemimethylated DNA hairpin D1 and the endonuclease GlaI, making a double-strand break into the fully methylated hairpin D2 that results in enhanced fluorescence. The methylated CpG site is located within the endonuclease GlaI recognition site (GCGm^5^C/Gm^5^CGm^5^C).

The Dnmt1 activity assay was carried out using a modified protocol [26] with separation of the stages of DNA hairpin methylation and its cleavage. The substrate used was the CpG-hemimethylated DNA hairpin D1 containing the Cy3 fluorophore and the BHQ2 quencher at the 5′ and 3′-ends, respectively. Methylation of the second CpG site of hairpin D1 results in DNA hairpin D2, which serves as a substrate for the double-strand break-inducing endonuclease GlaI. This results in the release of the 5-mer 3′- and 5′-terminal fragments and an increase in fluorescence (Figure 5).

Reaction mixture (50 µL) contained 30 nM D1, 1–5 µM SAM, or 20–100 µM of its phosphorus-containing analogue (rac-SAM-P_H_, (R,S)-SAM-P_H_, or rac-SAM-P_5_) in buffer A. Reactions were initiated by the addition of Dnmt1 (final concentration 10 nM), carried out at 37 °C for 4, 8, 16, or 24 h, and then stopped (95 °C, 2 min). Then MgCl_2_ (final concentration 5 mM) was added to the reaction mixture, and the DNA hairpin was cleaved with endonuclease GlaI (0.05 U/1.0 µL of the reaction mixture) at 37 °C for 40 min. Reactions were terminated (95 °C, 2 min), and after the addition of water (150 µL), transferred to a 96-well microplate to measure the fluorescence.

DNA hairpin D2 (methylation product) after its treatment with endonuclease GlaI was used as a control corresponding to 100% methylation. DNA hairpin D1, after its treatment with endonuclease GlaI in the presence of 5 mM MgCl_2_, was used as a control corresponding to 0% methylation. The methylation degree (*M*) was calculated as the ratio of cleaved and uncleaved DNA, using the formula:

$$M=\frac{I-I_{0}}{I_{100}-I_{0}}\;$$

where *I*, *I*_0_ и *I*_100_—fluorescence intensity in the reaction mixture, 0% control and 100% control, respectively.

Inhibitory activity of phosphorus-containing analogues of SAM and SAH was assayed following the same protocol in the reaction mixtures containing 5 µM SAM, and 100 µM of (R,S)-SAM-P_H_, rac-SAM-P_5_, rac-SAH-P_H_, or rac-SAH-P_5_ (37 °C, 24 h).

### 4.2. COMT Activity Assay and Inhibitory Activity of Phosphorus-Containing Analogues of SAH

Reaction mixtures (550 μL) in potassium phosphate buffer (50 mM, pH 7.5) contained protocatechuic aldehyde (3 mM), SAM or (R,S)-SAM-P_H_ (6 mM), COMT (20 μM), MgCl_2_ (18 mM), D_2_O (10 vol%) for the frequency lock, and either SAH, SAH-P_5_, or SAH-P_H_ (1.5 mM). The reaction mixtures were incubated at 37 °C in the NMR tubes. The formation of vanillin and isovanillin (Figure 1c) was monitored by the appearance of corresponding signals in ^1^H NMR spectra (Bruker (Billerica, MA, USA) AVANCE 600 MHz spectrometer). The concentration of the products was calculated by integration of the NMR signals relative to the internal standard sodium trimethylsilylpropanesulfonate (DSS-d_6_). All spectra were processed with MestReNova 14.2.2.

### 4.3. Statistical Analyzis

All data were obtained from at least five independent experiments. The data are presented as means ± standard deviations. Statistical significance was determined by one-way ANOVA, followed by Dunnett’s post hoc test using GraphPad Prism 9.5 (GraphPad Software, Boston, MA, USA). *p*-values ≤ 0.01 were considered significant.

## 5. Conclusions

The results of this study open up a new avenue for the chemical regulation of SAM-dependent enzymatic methylation using (R,S)-SAM-P_H_, which serves as a methyl group donor in the DNA methyltransferase reaction catalyzed by Dnmt3a but not Dnmt1. Obviously, (R,S)-SAM-P_H_ will not be equally well recognized by all methyltransferases, which makes the compound an interesting regulator of the methylation processes. Substrate properties of *L*-Met-P_H_ in the *S*-adenosylmethionine synthetase reaction [22] open up a possibility for using a Trojan-horse strategy to generate (R,S)-SAM-P_H_ in the cell and to develop novel dual-action inhibitors. Recently we demonstrated this possibility for *E. coli* using multi-target antibacterial distal *H*-phosphinic analogue of α-ketoglutarate, which was catabolized into the corresponding multi-target glutamate analogue with excellent antibacterial activity [51]. *L*-Met-P_H_ has very good fungicidal activity in vitro and excellent activity in field trials against rice blast disease [52], but it does not inhibit the growth of eukaryotic cells under standard conditions. Respectively, a properly designed transport form of *L*-Met-P_H_ must be used in the experiments with eukaryotic cells that will allow effective regulation of intracellular methylation processes.

## Data Availability

The original contributions presented in this study are included in the article/supplementary material. Further inquiries can be directed to the corresponding author.

## Supplementary Materials

The following supporting information can be downloaded at: https://www.mdpi.com/article/10.3390/ijms26178590/s1.
