# Supplementary material for: Phospho-Switch: Regulation of the Activity of SAM-Dependent Methyltransferases Using H-Phosphinic SAM Analogue
Source: Int J Mol Sci. 2025 Sep 4;26(17):8590. doi: 10.3390/ijms26178590 (PMC12428852; doi:10.3390/ijms26178590)
Supplement: Supplementary file 1 [file ijms-26-08590-s001.zip › ijms-3769538-supplementary.pdf]

Supplementary Material

Phospho-switch: Regulation of the activity of SAM-dependent methyltransferases using *H*-phosphinic SAM analogue

Vsevolod L. Filonov [1], Maxim A. Khomutov [1], Alexander Yu. Rudenko [2,3], Sofia S. Mariasina [2,3,4], Ratislav M. Ozhiganov [2,3,5], Alexander V. Sergeev [3], Sergei N. Kochetkov [1,3], Vladimir I. Polshakov [3], Elizaveta S. Gromova [3], Anastasia L. Khandazhinskaya [1] and Alex R. Khomutov [1,*]

[1] Engelhardt Institute of Molecular Biology, Russian Academy of Sciences, 119991 Moscow, Russia
[2] Belozersky Institute of Physico-Chemical Biology, Lomonosov Moscow State University, 119991 Moscow, Russia
[3] Faculty of Chemistry, Lomonosov Moscow State University, 119991 Moscow, Russia
[4] Institute of Pharmacy and Biotechnology, RUDN University, 117198 Moscow, Russia
[5] Higher Chemical College RAS, Mendeleev University of Chemical Technology, 125047 Moscow, Russia
* Correspondence: alexkhom@list.ru

This PDF file includes:
Figures S1 to S3

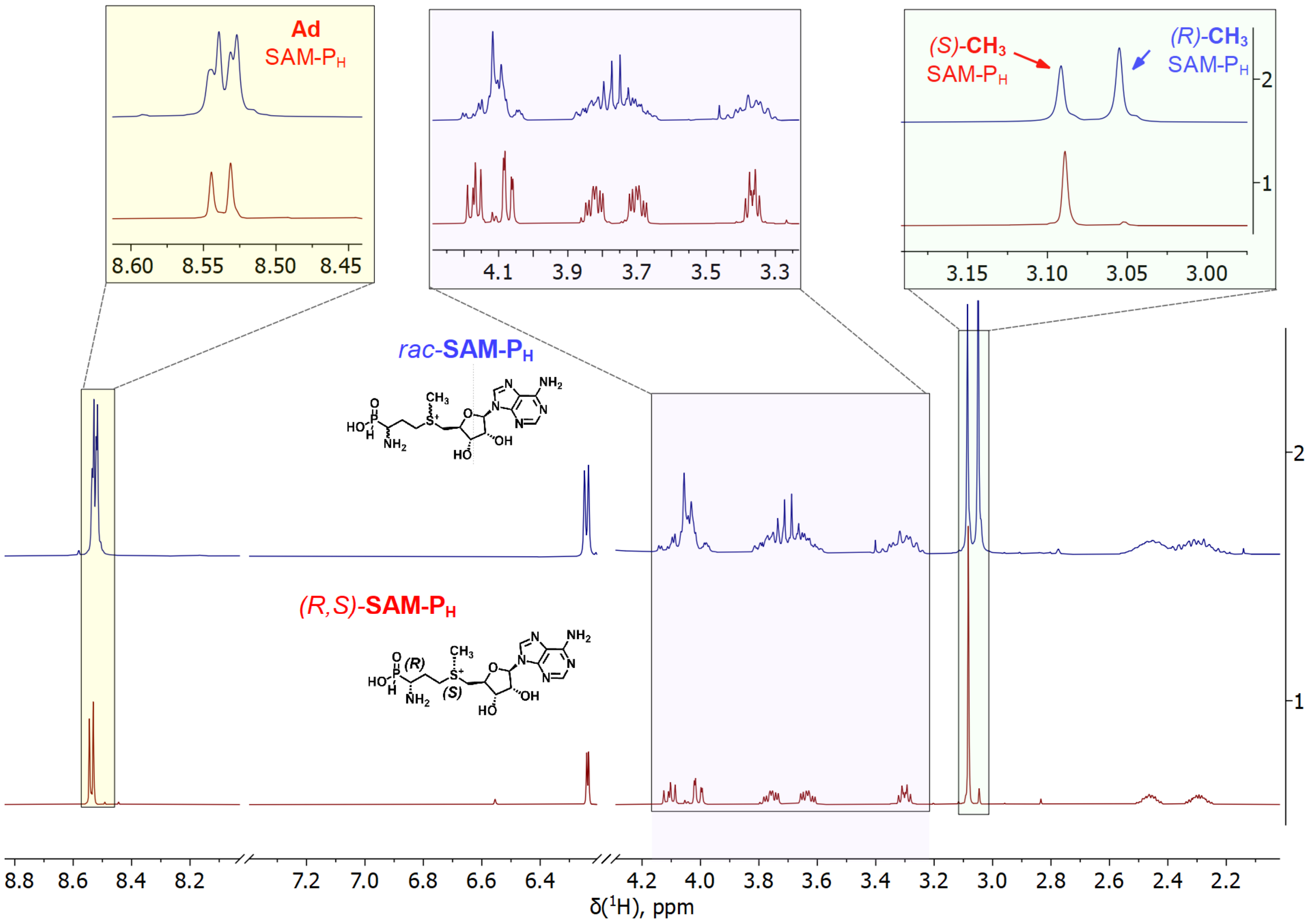

Supplementary Figure S1. ¹H NMR spectra of chemically synthesized *rac*-SAM-P_H and enzymatically synthesized (*R*,*S*)-SAM-P_H

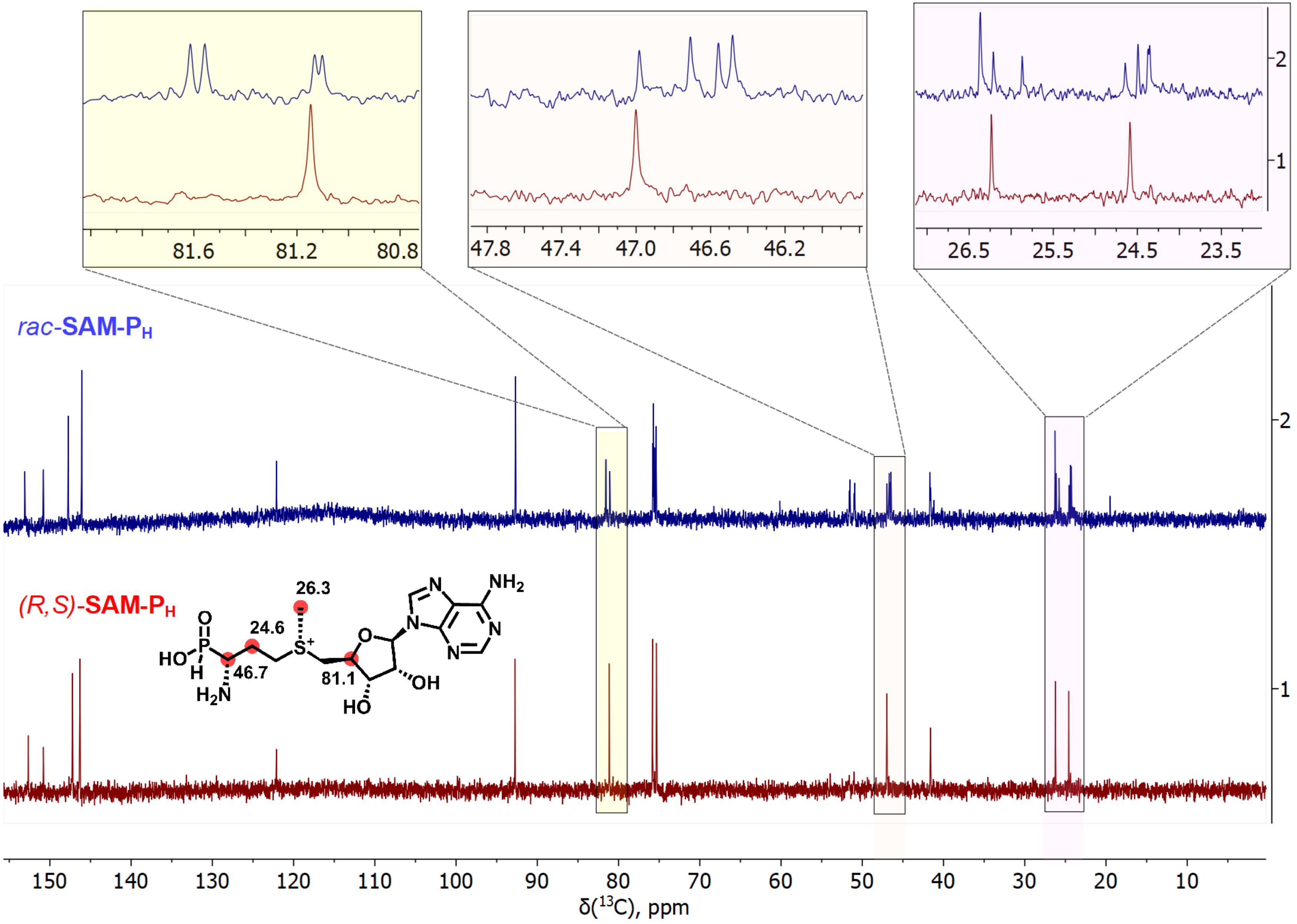

Supplementary Figure S2. ^{13}C NMR spectra of chemically synthesized *rac*-SAM-P$_H$ and enzymatically synthesized (*R*,*S*)-SAM-P$_H$

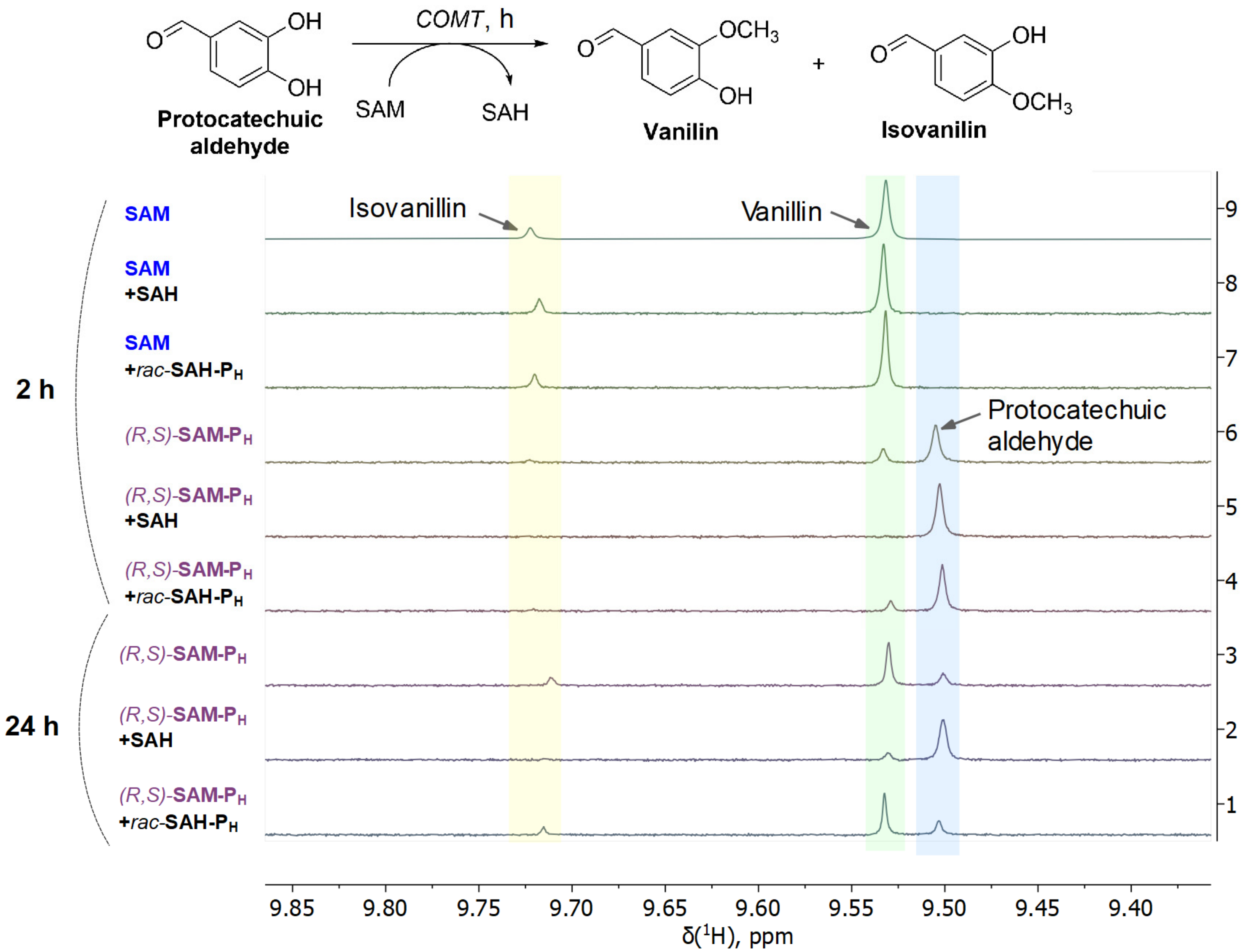

Supplementary Figure S3. Substrate properties of SAM, *rac*-SAM-P_H and (*R,S*)-SAM-P_H in COMT reaction and the inhibition of vanillin/isovanillin synthesis with SAH and *rac*-SAH-P_H (fragment of ^{1}H NMR spectra of reaction mixtures)
